# Variational Bayesian Pansharpening with Super-Gaussian Sparse Image Priors

**DOI:** 10.3390/s20185308

**Published:** 2020-09-16

**Authors:** Fernando Pérez-Bueno, Miguel Vega, Javier Mateos, Rafael Molina, Aggelos K. Katsaggelos

**Affiliations:** 1 Departamento de Ciencias de la Computación e Inteligencia Artificial, Universidad de Granada, 18071 Granada, Spain; jmd@decsai.ugr.es (J.M.); rms@decsai.ugr.es (R.M.); 2 Departamento de Lenguajes y Sistemas Informáticos, Universidad de Granada, 18071 Granada, Spain; mvega@ugr.es; 3 Department of Electrical Engineering and Computer Science, Northwestern University, Evanston, IL 60208-3118, USA; aggk@eecs.northwestern.edu

**Keywords:** pansharpening, variational Bayesian, image fusion, super-Gaussians

## Abstract

Pansharpening is a technique that fuses a low spatial resolution multispectral image and a high spatial resolution panchromatic one to obtain a multispectral image with the spatial resolution of the latter while preserving the spectral information of the multispectral image. In this paper we propose a variational Bayesian methodology for pansharpening. The proposed methodology uses the sensor characteristics to model the observation process and Super-Gaussian sparse image priors on the expected characteristics of the pansharpened image. The pansharpened image, as well as all model and variational parameters, are estimated within the proposed methodology. Using real and synthetic data, the quality of the pansharpened images is assessed both visually and quantitatively and compared with other pansharpening methods. Theoretical and experimental results demonstrate the effectiveness, efficiency, and flexibility of the proposed formulation.

## 1. Introduction

Remote sensing sensors simultaneously capture a Multispectral (MS) low resolution image along with a single-band high resolution image of the same area, referred to as Panchromatic (PAN) image. However, MS high-resolution images are needed by many applications, such as land use and land cover analyses or change detection. Pansharpening is a technique that fuses the MS and PAN images into an MS high resolution image that has the spatial resolution of the PAN image and the spectral resolution of the MS one.

In this paper we formulate the pansharpening problem following the Bayesian framework. Within this framework, we use the sensor characteristics to model the observation process as a conditional probability distribution. The observation process describes both the MS high resolution image to MS low resolution image relationship and how the PAN image is obtained from the MS high resolution one. This probability distributions provides fidelity to the observed data in the pansharpened image reconstruction process. together with from fidelity to the data, Bayesian methods incorporate prior knowledge on the MS high resolution image in the form of prior probability distributions. Crisp images, such as high resolution MS images, are expected to have Super-Gaussian (SG) statistics, while upsampled images suffer from blur that smooths out sharp gradients, making them more Gaussian in their statistics [1]. Our goal is to integrate the sharp edges of the PAN image into the pansharpened image, leading to less Gaussian statistics which makes SG priors a suitable choice. SG priors have been successfully applied to other image processing tasks, such as compressed sensing [2], blind deconvolution [1,3] and blind color deconvolution [4] and so it is also expected to produce good results in pansharpening. However, the form of the SG prior does not allow us to obtain the posterior distribution in an analytical way, making full Bayesian inference intractable. Hence, in this paper, we use the variational Bayesian inference to estimate the distribution of the pansharpened image as well as the model parameters from the MS low resolution and PAN images.

The rest of the paper is organized as follows: a categorization and short review of related pansharpening methods is presented in Section 2. In Section 3 the pansharpening problem is mathematically formulated. Following the Bayesian modelling and inference, in Section 4 we propose a fully Bayesian method for the estimation of all the problem unknowns and model parameters. In Section 5, the quality of the pansharpened images is assessed both visually and quantitatively and compared with other classic and state-of-the-art pansharpening methods. In Section 6 we discuss the obtained results and finally, Section 7 concludes the paper.

## 2. Related Work

Early pansharpening techniques, such as in the Brovey method [5], substituted some bands for image visualization or performed simple arithmetic transformations. Other classical methods included the transformation of the MS image and the substitution of one of its components by the high spatial resolution PAN image. Examples of this strategy are Principal Components Analysis PCA substitution [6], Brovey Transform [7] and Intensity-Hue-Saturation (IHS) [8] methods. A review of those early methods, among others, can be found in [9].

Over the past 20 years, numerous methods have been presented and, in an attempt to bring some order to the diversity of approaches, different reviews, comparisons and classifications have been proposed in the literature (see, for instance, [10,11,12,13,14,15,16,17]) each one with different criteria and, therefore, with a different categorization. Nevertheless, in the last years, there seems to be a consensus in three main categories, namely Component Substitution (CS), Multi-Resolution Analysis (MRA) and Variational Optimization (VO) [15,16,17]. Additionally, the increasing number of Deep Learning (DL)-based pansharpening methods proposed in recent years can be regarded as a new category.

The Component Substitution (CS) category includes the most widely used pansharpening methods. CS methods [12] usually upsample the MS image to the size of the PAN image and transform it to another space that separates the spatial and spectral image components. Then, the transformed component containing the spatial information is substituted by the PAN image (possibly, after histogram matching). Finally, the backward transform is applied to obtain the pansharpened image. Examples of these methods include the already mentioned PCA substitution [6], IHS methods [8,18,19], the Gram–Schmidt (GS) methods [20] and Brovey transform [7]. In [21], the transformation is replaced by any weighted average of the MS bands. It is shown that this approach generalizes any CS image fusion method. Determination of the weights has been carried out in different ways. For instance, in [22] the weights are optimally estimated to minimize the mean squared error while in [23] they are set to the correlation coefficient between a single band low resolution image (obtained from the MS image) and each MS band. A local criterion, based on the belonging of a given pixel to a fuzzy cluster, was applied in [24] to estimate weights that are different for each pixel of the image. To obtain a crisper MS high-resolution image, in [25] a Wiener deconvolution of the upsampled MS bands was performed before fusion.

In general, CS-based methods produce spectral distortions due to the different statistics of the PAN image and the transformed component containing the spatial details. To tackle this issue, Multi-Resolution Analysis (MRA) methods decompose the MS and PAN images to different levels, extract spatial details from the decomposed PAN image, and inject them into the finer scales of the MS image. This principle is also known as the ARSIS concept [10]. The High-Pass Filtering (HPF) algorithm in [11,18], can be considered to be the first approach in this category where only two levels are considered. Multi-scale decompositons, such as the wavelet transform (WT) [26,27,28], the Generalized Laplacian Pyramid (GLP) [29,30,31] or the Non-Subsampled Contourlet Transform (NSCT) [32,33,34], were used to bring more precision to the methods. The “a trous” wavelet transform (AWT) was the preferred decomposition technique [26,28] until the publication of [31] showed the advantages of GLP over AWT. This was later corroborated in [14] where a comparison of different methods based on decimated and undecimated WT, AWT, GLP and NSCT concluded that GLP outperforms AWT because it better removes aliasing. MRA category also includes the Smoothing Filter Based Intensity Modulation (SFIM) method [35,36], which first upsamples the MS image to the size of the PAN one and then uses a simplified solar radiation and land surface reflection model to increase its quality, and the Indusion method [37] in which upscaling and fusion steps are carried out together.

Deep Learning (DL) techniques have gained prominence in the past years and several methods have been proposed for pansharpening. As far as we know, the use of Deep Neural Networks (DNN) for pansharpening were first introduced in [38] where a Modified Sparse Denoising Autoencoder  (MSDA) algorithm was proposed. For the same task, a Coupled Sparse Denoising Autoencoder (CSDA) was used in [39]. Convolutional neural networks were introduced in [40] and also used, for instance, in [41]. Instead of facing the difficult task of learning the whole image, residual networks [42,43] learn, from upsampled MS and PAN patches, only the details of the MS high-resolution image that are not already in the upsampled MS image and add them to it to obtain the pansharpened image. To adjust the size of the MS image to the size of the PAN one in a coarse-to-fine manner, two residual networks in cascade were set in the so called Progressive Cascade Deep Residual Network (PCDRN)  [44]. In [45] a multi-scale approach is followed by learning a DNN to upsample each NSCT directional sub-band from the MS and PAN images. In general, the main weaknesses of the DL techniques are the high computational resources needed for training, the need of a huge amount of training data, which, in the case of pansharpening, might not be available, and the poor generalization to satellite images not used during training. The absence of ground-truth MS high-resolution images, needed for training these DL methods, is a problem pointed-out by [46] where a non-supervised generative adversarial network (Pan-GAN) was proposed. The GAN aims to generate pansharpened images that are consistent with the spectral information of the MS image while maintaining the spatial information of the PAN image. However, the generalization of this technique to satellite images different from the ones used for training is not clear. The adaptation of general image fusion methods, like the U2Fusion method in [47], to the pansharpening problem is a promising research area.

From a practical perspective, Variational Optimization (VO)-based methods present advantages both from a theoretical as well as computational points of view [48]. VO-based methods mathematically model the relation between the observed images and the original MS high resolution image, building an energy functional based on some desired properties of the original image. The pansharpened image is obtained as the image that minimizes this energy functional [49]. This mathematical formulation allows to rigorously introduce and process features that are visually important into the energy functional. Variational optimization can be considered as a particular case of the Bayesian approach [50], where the estimated image is obtained by maximizing the posterior probability distribution of the MS high resolution image. Bayesian methods for pansharpening formulate the relations between the observed images and the original MS high resolution image as probability distributions, model the desired properties as prior distributions and use Bayes’ theory to estimate the pansharpened image based on the posterior distribution of the original MS high resolution  image.

Following the seminal P+Xs method [51], the PAN image is usually modelled as a combination of the bands of the original high resolution mutispectral image. However, in [49] this model was generalized by substituting the intensity images by their gradients. Note that while the P+Xs method [51] preserves spectral information, it produces blurring artifacts. To remove blur while preserving spectral similarity, other restrictions are introduced as reasonable assumptions or prior knowledge about the original image such as Laplacian prior [52], total variation [53,54], sparse representations [55], band correlations [56,57], non-local priors [58,59], etc. Spectral information is also preserved by enforcing the pansharpened image to be close to the observed MS one when downsampled to the size of the latter [52,60,61]. A special class of VO-based methods are the super-resolution methods which model pansharpening as the inverse problem of recovering the original high-resolution image by fusing the MS image and the PAN (see [52,62] for a recent review and [63] for a recent work). Deconvolution methods, such as [64], also try to solve the inverse problem but the upsampling of the MS image to the size of the PAN one is performed prior to the pansharpening procedure. Registration and fusion are carried out simultaneously in [65].

Note that the variational Bayesian approach, also followed in this paper, is more general than variational optimization. While VO-based methods aim at obtaining a single estimate of the pansharpened image, the variational Bayesian approach estimates the whole posterior distribution of the pansharpened images and the model parameters, given the observations. When a single image is needed, the mode of the distribution is usually selected, but other solutions can be obtained, for instance, by sampling the estimated distribution. Even more, the proposed approach allows us to simultaneously estimate the model parameters along with the pansharpened image using the same framework.

## 3. Problem Formulation

Let us denote by y the MS high-resolution image hypothetically captured with an ideal high-resolution sensor with *B* bands $y_{b}$, b=1,…,B, of size p=m×n pixels, that is, $y={\left[y_{1}^{T},\ldots ,y_{B}^{T}\right]}^{T}$, where the superscript T denotes the transpose of a vector or matrix. Note that each band of the image is flattened into a column vector containing its pixels in lexicographical order. Unfortunately, this high-resolution image is not available in real applications. Instead, we observe an MS low-resolution image $Y={\left[Y_{1}^{T},\ldots ,Y_{B}^{T}\right]}^{T}$ with *B* bands $Y_{b}$ of size P=M×N pixels with M<m, N<n.

The bands in this image are flattened as well to express them as a column vector. The relation between each low-resolution band, $Y_{b}$, and its corresponding high-resolution one, $y_{b}$, is defined by

(1) $$\begin{array}{c} Y_{b}=DHy_{b}+n_{b}=By_{b}+n_{b}, \end{array}$$

where D is P×p decimation operator, H is a p×p blurring matrix, B=DH, and the capture noise $n_{b}$ is modeled as additive white Gaussian noise with variance $\beta_{b}^{-1}$.

A single band high-resolution PAN image covering a wide range of frequencies is also provided by the sensor. This PAN image x of size p=m×n is modelled as an spectral average of the unknown high-resolution bands $y_{b}$, as 

(2) $$\begin{array}{c} x=\sum_{b=1}^{B}\lambda_{b}y_{b}+v, \end{array}$$

where $\lambda_{b}>0$ are known quantities that depend on each particular satellite sensor, and the capture noise v is modeled as additive white Gaussian noise with variance $\gamma^{-1}$.

Once the image formation is formulated, let us use the Bayesian formulation to tackle the problem of recovering y, the MS high resolution image, using the observed Y, its degraded MS low resolution and PAN x.

## 4. Bayesian Modelling and Inference

We model the distribution of each low resolution image $Y_{b}$, b=1,…,B, following the degradation model in Equation (1) as a Gaussian distribution with mean $By_{b}$ and covariance matrix $\beta_{b}^{-1}I$. Then, the distribution of the observed image Y is modelled by

(3) $$\begin{array}{c} p\left(Y|y,\beta \right)=\prod_{b=1}^{B}N\left(Y_{b}|By_{b},\beta_{b}^{-1}I\right), \end{array}$$

with $\beta =\{\beta_{1},\ldots ,\beta_{b}\}$.

Analogously, using the degradation model in Equation (2), the distribution of the PAN image x is given by

(4) $$\begin{array}{c} p\left(x|y,\gamma \right)=N\left(x|\sum_{b=1}^{B}\lambda_{b}y_{b},\gamma^{-1}I\right). \end{array}$$

The starting point for Bayesian methods is to choose a prior distribution for the unknowns. In this paper, we use SG distributions as priors for the MS high resolution image as

(5) $$\begin{array}{c} p\left(y|\alpha \right)=\prod_{b=1}^{B}\prod_{\nu =1}^{J}p\left(y_{b\nu }|\alpha_{b\nu }\right)=\prod_{b=1}^{B}\prod_{\nu =1}^{J}\prod_{i=1}^{p}Z\left(\alpha_{b\nu }\right)\exp\left[-\alpha_{b\nu }\rho \left(y_{b\nu }\left(i\right)\right)\right], \end{array}$$

with $\alpha_{b\nu }>0$ and $\alpha =\{\alpha_{11}\ldots ,\alpha_{LB}\}$ and $Z(\alpha_{b\nu })$ is a partition function. In Equation (5), $y_{b\nu }=F_{\nu }y_{b}$ is a filtered version of the *b*-th band, $y_{b}$, where ${\left\{F_{\nu }\right\}}_{\nu =1}^{J}$ is a set of *J* high-pass filters, $y_{b\nu }\left(i\right)$ is the *i*-th pixel value of $y_{b\nu }$, and ρ(·) is a penalty function. The image priors are placed on the filtered image $y_{b\nu }$. It is well-known that the application of high-pass filters to natural images returns sparse coefficients. Most of the coefficients are zero or close to zero while only the edge related coefficients remain large. Sparse priors enjoy SG properties, heavier tails, more peaked and positive excess kurtosis compared to the Gaussian distribution. The distribution mass is located around zero, but large values have a higher probability than in a Gaussian distribution. For $p(y_{b\nu }|\alpha_{b\nu })$ in Equation (5) to be SG, ρ(·) has to be symmetric around zero and the function $\rho (\sqrt{s})$ increasing and concave for s∈(0,∞). This condition is equivalent to $\rho^{\prime }\left(s\right)/\left|s\right|$ being decreasing on (0,∞), and allows ρ to be represented as

(6) $$\begin{array}{c} \rho \left(y_{b\nu }\left(i\right)\right)=\underset{\eta_{b\nu }\left(i\right)>0}{\inf}\;\frac{1}{2}\;\eta_{b\nu }\left(i\right)\;y_{b\nu }^{2}\left(i\right)\;-\rho^{*}\left(\frac{1}{2}\eta_{b\nu }\left(i\right)\right)\; \end{array}$$

(7) $$\begin{array}{c} \Rightarrow \rho \left(y_{b\nu }\left(i\right)\right)\leq L\left(y_{b\nu }\left(i\right),\eta_{b\nu }\left(i\right)\right)=\;\frac{1}{2}\;\eta_{b\nu }\left(i\right)\;y_{b\nu }^{2}\left(i\right)-\rho^{*}\left(\frac{1}{2}\eta_{b\nu }\left(i\right)\right)\;, \end{array}$$

where inf denotes the infimum, $\rho^{*}\left(\cdot \right)$ is the concave conjugate of ρ(·) and $\eta_{b\nu }={\left\{\eta_{b\nu }\left(i\right)\right\}}_{i=1}^{p}$ are a set of positive parameters. The relationship dual to Equation (6) is given by [66]

(8) $$\begin{array}{c} \rho^{*}\left(\frac{1}{2}\eta_{b\nu }\left(i\right)\right)=\underset{y_{b\nu }\left(i\right)}{\inf}\;\frac{1}{2}\;\eta_{b\nu }\left(i\right)\;y_{b\nu }^{2}\left(i\right)-\rho \left(y_{b\nu }\left(i\right)\right)\;. \end{array}$$

To achieve sparsity, the function ρ should suppress most of the coefficients in $y_{b\nu }$ and preserve a small number of key features. Table 1 shows some penalty functions, corresponding to SG distributions (see [1]).

From Equations (3)–(5), the joint probability distribution p(Θ,Y,x), with Θ={y,β,γ,α} the set of all unknowns, is given by

(9) $$\begin{array}{c} p(\Theta ,Y,x)=p(Y|y,\beta )p(x|y,\gamma )p(y|\alpha )p(\beta )p(\gamma )p(\alpha ), \end{array}$$

where flat hyperpriors p(β), p(γ) and p(α) on the model hyperparameters have been included.

Following the Bayesian paradigm, inference will be based on p(Θ|Y,x). Since this posterior distribution cannot be analytically calculated due to the form of the SG distribution, in this paper we use the mean-field variational Bayesian model [67] to approximate p(Θ|Y,x) by the distribution q(Θ) of the form $q\left(\Theta \right)=\prod_{\theta \in \Theta }q\left(\theta \right)$, that minimizes the Kullback–Leibler divergence [68] defined as

(10) $$\begin{array}{cc} \mathrm{KL}\left(q(\Theta )\;||\;p(\Theta |Y,x)\right) & =\int q\left(\Theta \right)\log\frac{q(\Theta )}{p(\Theta ,Y,x)}\;d\Theta +\log p\left(Y\right)+\log p\left(x\right). \end{array}$$

The Kullback–Leibler divergence is always non-negative and it is equal to zero if and only if q(Θ)=p(Θ|Y,x).

Even with this factorization, the SG prior for y hampers the evaluation of this divergence, but the quadratic bound for ρ in Equation (7) allows us to bound the prior in Equation (5) with a Gaussian form such that

(11) $$\begin{array}{cc} \; & p\left(y_{b\nu }\left(i\right)|\alpha_{b\nu }\right)\geq Z\left(\alpha_{b\nu }\right)\exp\left[-\alpha_{b\nu }L\left(y_{b\nu }\left(i\right),\eta_{b\nu }\left(i\right)\right)\right],\;\;\forall \eta_{b\nu }\left(i\right)>0\;. \end{array}$$

We then define the lower bound of the prior $M_{\nu }\left(y_{\nu },\eta_{\nu }|\alpha_{\nu }\right)=\prod_{b}{M_{\nu }}_{b}\left(y_{b\nu },\eta_{b\nu }|\alpha_{b\nu }\right)$ where

(12) $$\begin{array}{c} {M_{\nu }}_{b}\left(y_{b\nu },\eta_{b\nu }|\alpha_{b\nu }\right)=\prod_{i=1}^{p}Z\left(\alpha_{b\nu }\right)\exp\left[-\alpha_{b\nu }L\left(y_{b\nu }\left(i\right),\eta_{b\nu }\left(i\right)\right)\right] \end{array}$$

and obtain the lower bound of the joint probability distribution

(13) $$\begin{array}{c} F\left(\Theta ,Y,x,\eta \right)=p\left(Y|y,\beta \right)p\left(x|y,\gamma \right)\prod_{\nu =1}^{J}M_{\nu }\left(y_{\nu },\eta_{\nu }|\alpha_{\nu }\right) \end{array}$$

to obtain the inequality logp(Θ,Y,x)≥logF(Θ,Y,x,η).

Utilizing the lower bound F(Θ,Y,x,η) for the posterior probability distribution in Equation (10) we minimize $\mathrm{KL}\left(q(\Theta )\;||\;F(\Theta ,Y,x,\eta )\right)$ instead of $\mathrm{KL}\left(q(\Theta )\;||\;p(\Theta |Y,x)\right)$.

As shown in [67], for each unknown θ∈Θ, the estimated q(θ) will have the form

(14) $$q\left(\theta \right)\propto \exp{\langle \log F(\Theta ,Y,x,\eta )\rangle }_{q(\Theta \setminus \theta )}\;,$$

where Θ∖θ represents all the variables in Θ except θ and ${\langle \cdot \rangle }_{q(\Theta \setminus \theta )}$ denotes the expected value calculated using the distribution q(Θ∖θ). When point estimates are required $\hat{\theta }={\langle \theta \rangle }_{q(\theta )}$ is used.

For variables with a degenerate posterior approximation, that is, for θ∈{β,γ,α}, the value where the posterior degenerates is given by [67]

(15) $$\begin{array}{c} \hat{\theta }=\arg\max_{\theta }{\langle \log F(\Theta ,Y,x,\eta )\rangle }_{q(\Theta \setminus \theta )}. \end{array}$$

Let us now obtain the analytic expressions for each unknown posterior approximation.

### 4.1. High Resolution Multispectral Image Update

Using Equation (14) we can show in a straightforward way that the posterior distribution for the high resolution MS image will have the form

(16) $$\begin{array}{c} q\left(y\right)=N\left(y|\langle y\rangle ,\Sigma_{y}\right)\;, \end{array}$$

where the inverse of the covariance matrix is given by

(17) $$\begin{array}{cc} \Sigma_{y}^{-1}= & \;\;\mathrm{diag}\left(\beta \right)⊗B^{T}B+\gamma \left(\lambda \lambda^{T}\right)⊗I_{p\times p}\\ \; & +\sum_{\nu }\left(\begin{array}{cccc} \alpha_{1\nu }F_{\nu }^{T}\mathrm{diag}\left(\eta_{1\nu }\right)F_{\nu } & 0_{p\times p} & \ldots & 0_{p\times p}\\ 0_{p\times p} & \alpha_{2\nu }F_{\nu }^{T}\mathrm{diag}\left(\eta_{2\nu }\right)F_{\nu } & \ldots & 0_{p\times p}\\ \vdots & \vdots & \ddots & \vdots \\ 0_{p\times p} & 0_{p\times p} & \ldots & \alpha_{B\nu }F_{\nu }^{T}\mathrm{diag}\left(\eta_{B\nu }\right)F_{\nu } \end{array}\right)\;, \end{array}$$

with ⊗ denoting the Kronecker product, diag(·) is a diagonal matrix formed from the elements of a vector and the mean is obtained as

(18) $$\begin{array}{c} \Sigma_{y}^{-1}\langle y\rangle =\left(\mathrm{diag}\left(\beta \right)⊗B^{T}\right)Y+\gamma \left(\mathrm{diag}\left(\lambda \right)⊗I_{p\times p}\right){\left(x^{T},x^{T},\ldots ,x^{T}\right)}^{T}\;. \end{array}$$

### 4.2. Variational Parameters Update

To estimate the value of the variational parameters, η introduced in Equation (7), we need to solve, for each band b∈{1,…,B}, filter ν∈{1,…,J}, and pixel i∈{1,…,p}, the optimization problem

(19) $$\begin{array}{cc} \;{\hat{\eta }}_{b\nu }\left(i\right) & =\arg\min_{\eta_{b\nu }\left(i\right)}{\langle L\left(y_{b\nu }\left(i\right),\eta_{b\nu }\left(i\right)\right)\rangle }_{q(y)}\\ \; & =\arg\min_{\eta_{b\nu }\left(i\right)}\frac{1}{2}\;\eta_{b\nu }\left(i\right)\;u_{b\nu }^{2}\left(i\right)\;-\rho^{*}\left(\frac{1}{2}\eta_{b\nu }\left(i\right)\right), \end{array}$$

where $u_{b\nu }\left(i\right)=\sqrt{\langle y_{b\nu }^{2}\left(i\right)\rangle }$. Since

(20) $$\begin{array}{cc} \rho^{*}\left(\frac{{\hat{\eta }}_{b\nu }\left(i\right)}{2}\right) & =\min_{x}\frac{1}{2}{\hat{\eta }}_{b\nu }\left(i\right)x^{2}-\rho \left(x\right) \end{array}$$

whose minimum is achieved at $x=u_{b\nu }\left(i\right)$, we have, differentiating the right hand side of (19) with respect to *x*,

(21) $$\begin{array}{c} {\hat{\eta }}_{b\nu }\left(i\right)=\rho^{\prime }\left(u_{b\nu }\left(i\right)\right))/u_{b\nu }\left(i\right). \end{array}$$

### 4.3. Model Parameters Update

The estimates of the noise variance in the degradation models in Equations (3) and (4) are obtained using Equation (15) as

(22) $$\begin{array}{cc} {\hat{\beta }}_{b}^{-1} & =\frac{\mathrm{tr}{\langle \left(Y_{b}-By_{b}\right){\left(Y_{b}-By_{b}\right)}^{T}\rangle }_{q(\Theta )}}{P},\;b=1,\ldots ,B, \end{array}$$

(23) $$\begin{array}{cc} {\hat{\gamma }}^{-1} & =\frac{\mathrm{tr}{\langle \left(x-\sum_{b=1}^{B}\lambda_{b}y_{b}\right){\left(x-\sum_{b=1}^{B}\lambda_{b}y_{b}\right)}^{T}\rangle }_{q(\Theta )}}{p}\;, \end{array}$$

where tr(·) represents the trace of the matrix.

From Equation (14) we obtain the following distribution for the parameter $\alpha_{b\nu }$ of the SG prior in Equation (5).

(24) $$\begin{array}{c} q\left(\alpha_{b\nu }\right)=const+\sum_{i=1}^{p}\log Z\left(\alpha_{b\nu }\right)\exp\left[-\alpha_{b\nu }\rho \left(y_{b\nu }\left(i\right)\right)\right]. \end{array}$$

The mode of this distribution can be obtained (see [69]) by solving

(25) $$\begin{array}{c} \frac{\partial Z({\hat{\alpha }}_{b\nu })}{\partial {\hat{\alpha }}_{\nu s}}=\frac{\mathrm{tr}\left(\left(F_{\nu }^{T}F_{\nu }\right)\langle y_{b\nu }y_{b\nu }^{T}\rangle \right)}{p}. \end{array}$$

The $\ell_{p}$ penalty function shown in Table 1 produces proper priors, for which the partition function can be evaluated, but the log penalty function produces an improper prior. We tackle this problem examining, for $\alpha_{b\nu }\neq 1$, the behavior of

(26) $$\begin{array}{c} {Z(\alpha_{b\nu },K)}^{-1}=\int_{-K}^{K}\exp\left[-\alpha_{b\nu }\rho \left(t\right)\right]dt \end{array}$$

and keeping in $\partial Z\left(\alpha_{b\nu }\right)/\partial \alpha_{b\nu }$ the term that depends on $\alpha_{b\nu }$. This produces for the log prior

(27) $$\begin{array}{c} \frac{\partial Z({\hat{\alpha }}_{b\nu })}{\partial {\hat{\alpha }}_{b\nu }}={\left({\hat{\alpha }}_{b\nu }-1\right)}^{-1}. \end{array}$$

### 4.4. Calculating the Covariance Matrices

The matrix $\Sigma_{y}$ in Equation (17) must be explicitly computed to find its trace and also to calculate ${\hat{\eta }}_{b\nu }\left(i\right)$. However, since its calculation is very intense, we propose the following approximation. We  first approximate $\mathrm{diag}(\eta_{b\nu })$ using

(28) $$\mathrm{diag}\left(\eta_{b\nu }\right)\approx z\left(\eta_{b\nu }\right)I_{p\times p},$$

where $z(\eta_{b\nu })$ is calculated as the mean of the values in $\eta_{b\nu }$.

We then use the approximation

$$\begin{array}{c} \Sigma_{y}^{-1}\approx \left(\begin{array}{cccc} \Sigma_{y_{1}}^{-1} & 0_{p\times p} & \ldots & 0_{p\times p}\\ 0_{p\times p} & \Sigma_{y_{2}}^{-1} & \ldots & 0_{p\times p}\\ \vdots & \vdots & \ddots & \vdots \\ 0_{p\times p} & 0_{p\times p} & \ldots & \Sigma_{y_{B}}^{-1} \end{array}\right) \end{array}$$

with

$$\begin{array}{cc} \; & \Sigma_{y_{b}}^{-1}\approx \beta_{b}B^{T}B+\gamma \lambda_{b}^{2}I_{p\times p}+\sum_{\nu }\alpha_{b\nu }z\left(\eta_{b\nu }\right)F_{\nu }^{T}F_{\nu }=C_{b},\;b=1,\ldots ,B. \end{array}$$

Finally we have

$$\begin{array}{c} \langle y_{b\nu }^{2}\left(i\right)\rangle \approx {\left(\langle y_{b\nu }\left(i\right)\rangle \right)}^{2}+\frac{1}{p}\mathrm{tr}\left[C_{b}^{-1}F_{\nu }^{T}F_{\nu }\right]. \end{array}$$

### 4.5. Proposed Algorithm

Based on the previous derivations, we propose the Variational Bayesian SG Pansharpening Algorithm in Algorithm 1. The linear equations problem in Equation (18), used in step 4 of Algorithm 1, has been solved using the Conjugate Gradient approach.
**Algorithm 1:** Variational Bayesian SG pansharpening.**Require:** Observed multispectral image, Y, panchromatic image x, and λ parameter. Set $\Sigma_{y}^{\left(0\right)}=0$ and n=0. ${\langle y_{b}\rangle }^{\left(0\right)}$ is obtained by bicubic interpolation of $Y_{b}$, ∀b=1,…,B. **while** convergence criterion is not met **do**  1. Set n=n+1.  2. Obtain $\beta^{\left(n\right)}$, $\gamma^{\left(n\right)}$ and $\alpha_{b\nu }^{\left(n\right)}$ from Equations (22), (23) and (25) respectively.  3. Using ${\langle y\rangle }^{\left(n-1\right)}$ and $\Sigma_{y}^{\left(n-1\right)}$, update the variational parameters ${\hat{\eta }}_{b\nu }^{\left(n\right)}$, ∀b,ν from Equation (21).  4. Using $\beta^{\left(n\right)}$, $\gamma^{\left(n\right)}$, $\alpha_{b\nu }^{\left(n\right)}$, and ${\hat{\eta }}_{b\nu }^{\left(n\right)}$, update $\Sigma_{y}^{-1(n)}$ in Equation (17) and solve Equation (18) for ${\langle y\rangle }^{\left(n\right)}$. **end while** Output the high resolution hyperspectral image $\hat{y}={\langle y\rangle }^{\left(n\right)}$.

## 5. Materials and Methods

**Figure 1:**
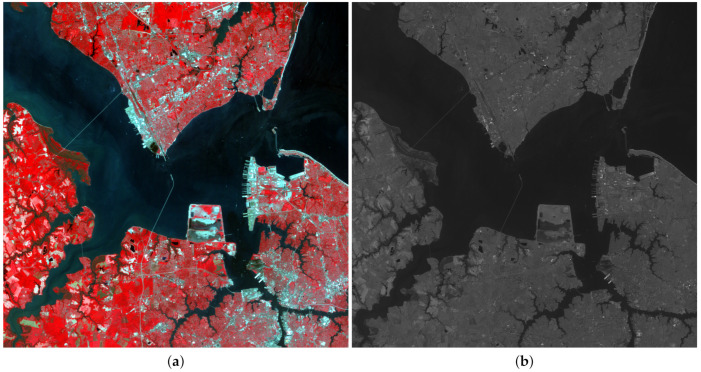
Observed LANDSAT 7-ETM+ Chesapeake Bay image: (**a**) 1024×1024 multispectral (MS), (**b**) 2048×2048 panchromatic (PAN).

**Figure 2:**
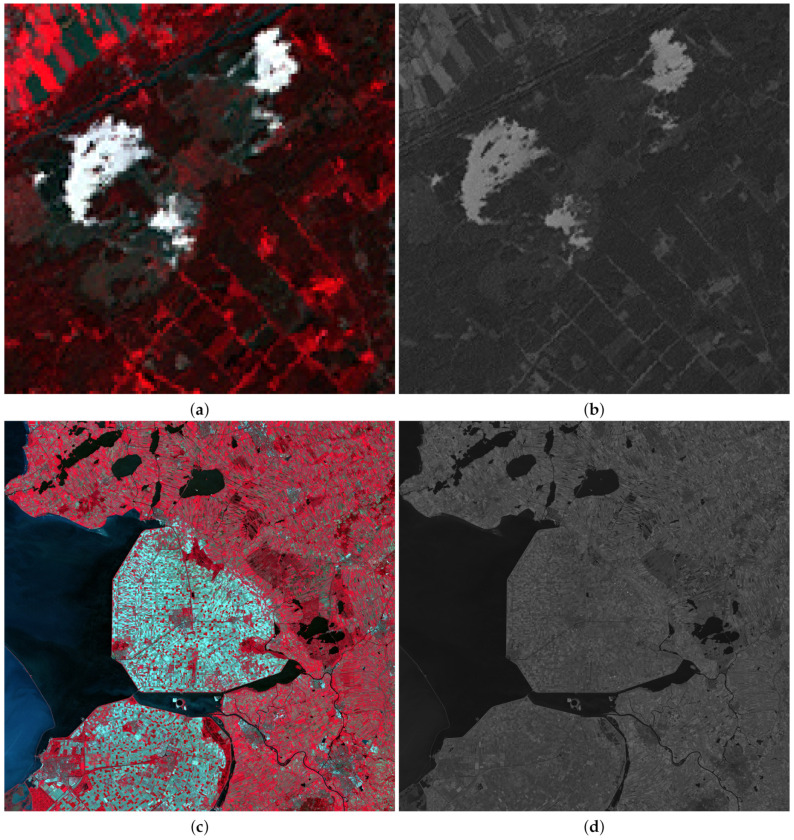
Observed LANDSAT 7-ETM+ Netherland images: (**a**) 128×128 MS, (**b**) 256×256 PAN, (**c**) 2048×2048 MS, (**d**) 4096×4096 PAN.

**Figure 3:**
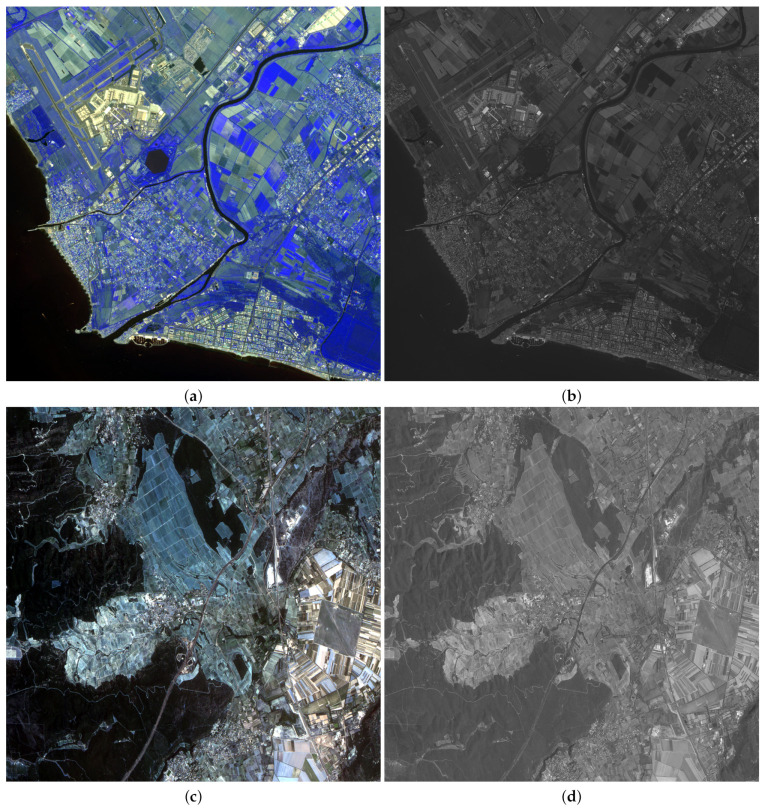
Observed SPOT-5 Roma image: (**a**) 1024×1024 MS, (**b**) 4096×4096 PAN. FORMOSAT-2 Salon-de-Provence image: (**c**) 1024×1024 MS, (**d**) 4096×4096 PAN.

To test the performance of the proposed methodology on different kind of images, five satellite images were used: three LANDSAT 7-ETM+ [70] images, a SPOT-5 [71] image and a FORMOSAT-2 [72] image. LANDSAT MS images have six bands and a ratio between PAN and MS images p/P=4. Figure 1 and Figure 2 show RGB color images formed by the bands B4, B3 and B2 of LANDSAT MS images, and their corresponding PAN images. Figure 1 corresponds to an area from Chesapeake Bay (US) while Figure 2 depicts two areas from Neatherland.SPOT-5 MS images have four bands and two PAN images, with resolution ratios of p/P=4 and p/P=16, are provided. FORMOSAT-2 MS images also have four bands and a ratio between PAN and MS images p/P=16. Figure 3a,c show the RGB color images formed from bands B3, B2 and B1 bands of a SPOT-5 image from Roma (IT) and a FORMOSAT-2 MS image from Salon-de-Provence (FR) and Figure 3b,d their corresponding PAN images.

**Figure 4:**
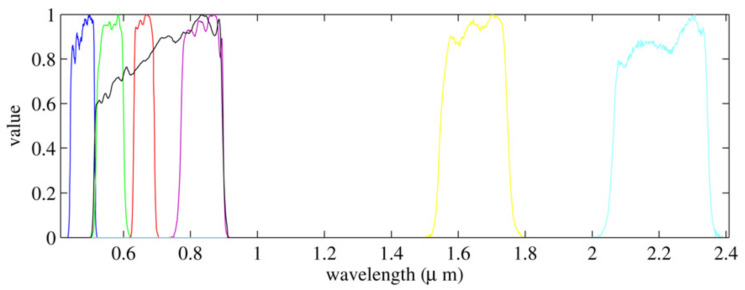
LANDSAT 7-ETM+ band spectral response normalized to one.

Both the observed Y and x images have been normalized to the range [0,1] before running Algorithm 1. The convergence criterion in the algorithm was $\|{\langle y\rangle }^{\left(n\right)}-{\langle y\rangle }^{\left(n-1\right)}{\|}^{2}/{\left\|{\langle y\rangle }^{\left(n\right)}\right\|}^{2}\leq 10^{-6}$ or 50 iterations were reached, whatever occurs first. The relationship between the MS high resolution image and the panchromatic image in Equation (2) is governed by the parameters λ that need to be set before pansharpening is carried out. If we knew the sensor spectral response characteristics, the values of λ could be estimated from them. For instance, for LANDSAT 7-ETM+, Figure 4 shows the sensor spectral response curves for the MS bands B1-B6, shown in color, and the PAN band shown in black. For this sensor, the PAN band mainly overlaps B2-B4 MS bands, and λ coefficients could be obtained from this overlapping (see [52]). In this paper, however, a more general approach is followed to estimate λ from the observations. First, we define X=Dx, a version of the PAN image downsampled to the size of the MS image. Then, since the sensor spectral response is the same in high and low resolution, the parameters λ can be obtained by solving

(29) $$\begin{array}{cc} \; & \lambda =\underset{\lambda }{\mathrm{argmin}}{\left\|X-\sum_{b=1}^{B}\lambda_{b}Y_{b}\right\|}^{2}, \end{array}$$

(30) $$\begin{array}{cc} \; & \mathrm{subject}\;\mathrm{sto}\lambda_{b}\geq 0,\;\forall b,\;\sum_{b=1}^{B}\lambda_{b}=1\;. \end{array}$$

Table 2 shows the λs associated to the different considered observed images. For the LANDSAT 7-ETM+ images only the first four bands are positive and $\lambda_{5}$ and $\lambda_{6}$ values are 0 since we know that bands B5 and B6 are not covered by the panchromatic sensor. For this process, each band is normalized to the interval [0,1]. Note that due to the normalization, the estimated λ values do not only depend on the sensor spectral response but also on the observed area characteristics. This explains the differences between the obtained λ values for the images in Figure 2a,c. Although those images are from the same area of Netherlands, clouds in Figure 2a modify the estimation of the values of λ.

## 6. Discussion

Within the variational Bayesian methodology, two methods are proposed in this paper: one using the log penalty function (see Table 1), hence, named log method, and another using the $\ell_{p}$ penalty function, with p=1, referred as ℓ1 method. The proposed methods have been compared with the following classic and state-of-the-art pansharpening methods: the Principal Component Analysis (PCA) [6], the Intensity–Hue–Saturation (IHS) transform [19], the Brovey transform (Brovey) [7], the Band-Dependent Spatial-Detail (BDSD) method in [22], the Gram-Schmidt (GS) method in [20], the Gram-Schmidt adaptive (GSA) method in [21], the Partial Replacement Adaptive Component Substitution (PRACS) method in [23], the High Pass Filtering (HPF) algorithm in [18], the Smoothing Filter Based Intensity Modulation (SFIM) method [35,36], the Indusion method in [37], the Additive A Trous Wavelet Transform (ATWT) in [26], the Additive Wavelet Luminance Proportional (AWLP) method in [28], the ATWT Model 2 (ATWT-M2) and ATWT Model 3 (ATWT-M3) methods in [10], the Generalized Laplacian Pyramid (GLP)-based methods in [29], concretely the modulation transfer functions (MTF)-GLP, GLP with High Pass Modulation (MTF-GLP-HPM), and GLP with Context Based Decision (MTF-GLP-CBD) methods, and the pansharpening method using a Total Variation (TV) image model in [53]. We have used the implementation of the methods and measures provided by the Pansharpening Toolbox (https://rscl-grss.org/coderecord.php?id=541) [13]. For those methods not included in the toolbox we have used the code provided by the authors. The code of the proposed methods will be publicly available at https://github.com/vipgugr. We have also included the results of bilinear interpolating the MS image to the size of the PAN, marked as EXP, as a reference. Both quantitative and qualitative comparisons of the different methods have been performed.

### 6.1. Quantitative Comparison

A common problem in pansharpening is the nonexistence of a MS high resolution ground-truth image to compare with. Hence we performed two kinds of quantitative comparisons. Firstly, the images obtained using the different methods have been compared following Wald’s protocol [73] as follows: the observed MS image, Y, and the PAN image, x, are downsampled by applying the operator D to generate low resolution versions of them. Then, pansharpening is applied to those low resolution images and the obtained estimation of the MS image, $\hat{y}$, is quantitatively compared with the observed MS image, Y. Secondly, the different methods have been compared using Quality with No Reference (QNR) measures [13,74]. As previously stated, for the LANDSAT image in Figure 1, the resolution ratio between MS and PAN images is p/P=4. Since the SPOT-5 satellite provides two PAN images, two experiments were carried out on the image in Figure 3, one with a decimation ratio of 4 and another with a ratio of 16. For the FORMOSAT-2 image the ratio is p/P=16. However, for the sake of completeness, two experiments were also carried out, one assuming a decimation ratio of 4 and another with a ratio of 16.

Both spatial and spectral quality metrics have been used to compare the results obtained using the different methods. Details for the metrics used is shown below:

Spatial measures:

- Q
  – Universal Quality Index (UQI) [75] averaged on all MS bands.
  – Range: [-1, 1]
  – The higher the the better.
- Q4, Q8
  – Instances of the $Q2^{n}$ [76] index taking values. Suitable to measure quality for multiband images having an arbitrary number of spectral bands. Q4 is used for SPOT-5 and FORMOSAT-2 images which have four bands and Q8 for the LANDSAT image with six bands.
  – Range: [0, 1]
  – The higher the better.
- Spatial Correlation Coefficient (SCC) [77]
  – Measures the correlation coefficient between compared images after the application of a Sobel filter.
  – Range: [0, 1]
  – The higher the better.
- QNR spatial distortion ($D_{S}$) [78]
  – Measures the spatial distortion between MS bands and PAN image.
  – Range: [0, 1]
  – The lower the better.

Spectral measures:

- Spectral Angle Mapper (SAM) [79]
  – For spectral fidelity. Measures the mean angle between the corresponding pixels of the compared images in the space defined by considering each spectral band as a coordinate axis
  – Range: [0, 180]
  – The lower the better.
- Erreur Relative Globale Adimensionnelle de Synthese (ERGAS) [80]
  – Measures spectral consistency between compared images.
  – Range: [0,∞[
  – The lower ERGAS value the better consistency, specially for values lower than the number of image bands *B*.
- QNR spectral distortion ($D_{\lambda }$) [78]
  – This measure is derived from the differences between the inter-band Q index values computed for HR and LR images.
  – Range: [0, 1]
  – The lower, the better.

Spatial and spectral measures:

- Jointly Spectral and Spatial Quality Index (QNR) [78]
  – QNR is obtained as the product of (1-$D_{S}$) and (1-$D_{\lambda }$).
  – Range: [0, 1]
  – The higher the better.

Table 3 shows the obtained figures of merit using Wald’s protocol for the LANDSAT image in Figure 1. As it is clear from the table, *ℓ*1 outperforms all the other methods both in spectral fidelity and the incorporation of spatial details. Note the high SCC value (meaning that the details in the PAN image have been successfully incorporated into the pansharpened image) while also obtaining the lowest spectral distortion as evidenced by the SAM and ERGAS values. The TV method obtains the second best results except for the SAM metric, for this metric, the proposed log method has the second best value. This method also obtains the third best values for ERGAS and SCC measures. GLP based and PRACS methods also obtain high values for the Q, Q8 indices and low value for SAM. However, their ERGAS and SCC performance is worse. Table 4 shows the QNR quantitative results for the LANDSAT image in Figure 1. In this table, the proposed methods achieve competitive results. Log obtains the best $D_{\lambda }$ value and this method together with *ℓ*1 obtain second and third QNR scores, respectively. Note that EXP obtained the highest score using QNR since bilinear interpolation of the observed MS low resolution image is used as the MS high resolution estimation to calculate $D_{S}$ and $D_{\lambda }$ calculations.

Table 5 and Table 6 show the quantitative results using Wald’s protocol for the LANDSAT images in Figure 2a,c, respectively. PRACS outperforms all other methods on the image in Figure 2a (see Table 5) and the proposed *ℓ*1 and log obtain the first and second best scores on the image in Figure 2c (see Table 6). Table 7 and Table 8 show the obtained QNR figures of merit for those two images. The proposed methods produce good $D_{S}$, $D_{\lambda }$ and QNR values for both images, both above 0.9 which supports their good performance. Again the EXP results are the best in all the measures for Table 8 and provides the best $D_{\lambda }$, for the image associated to Table 7. The *ℓ*1 method obtains the best $D_{S}$ for this image and BDSD the highest QNR.

**Figure 5:**
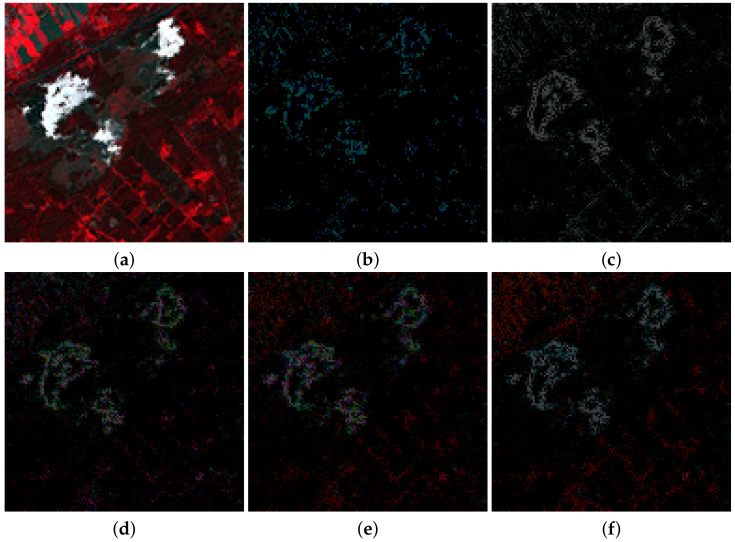
(**a**) Ground truth 128×128 image from Figure 2a. The normalized maximum absolute error minus the absolute error images images for the following methods, (**b**) Partial Replacement Adaptive Component Substitution (PRACS), (**c**) modulation transfer functions (MTF)-generalized Laplacian pyramid (GLP)-context based decision (CBD), (**d**) Total Variation (TV), (**e**) *ℓ*1 and (**f**) log.

**Figure 6:**
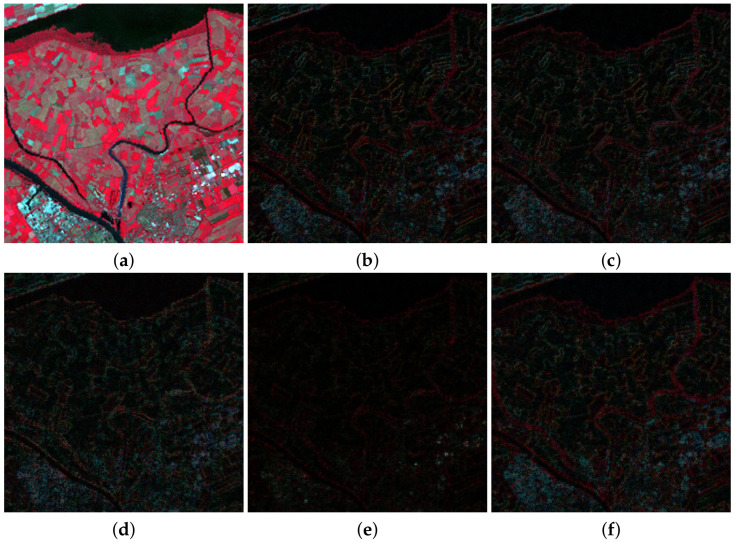
(**a**) Ground truth 256×256 image from Figure 2c. The normalized maximum absolute error images for the following methods: (**b**) PRACS, (**c**) MTF-GLP-CBD, (**d**) TV, (**e**) *ℓ*1 and (**f**) log.

Figure 5 and Figure 6 show a zoomed in region of the RGB color images formed by bands B4, B3, and B2 of MS ground truth images used to apply Wald’s protocol and also the absolute error images for the methods in Table 7 and Table 8. In those images, the darker the intensity the lower the absolute error. Figure 5 and Figure 6 are consistent with the quantitative comparison shown in Table 5 and Table 6, respectively. The best results for the image in Figure 2a were obtained using PRACS, while for the image in Figure 2c the best performing method is *ℓ*1. Note that brighter areas in Figure 5e,f correspond to the borders of cloudy areas in Figure 2a. We argue that since clouds alter the weights of λ estimated using Equation (30), the boundaries of clouds and land areas in Figure 2a are not well resolved. This explains a worse behavior of the proposed methods in the cloudy areas of this image.

Table 9 and Table 10 show, respectively, the quantitative results using Wald’s protocol for the SPOT-5 and the FORMOSAT-2 images in Figure 3 for the decimation ratios p/P=4 and p/P=16. The proposed log obtains the best figures of merit for the SPOT image in Figure 3a with p/P=4 except for Q and Q4 metrics. The Q values obtained by log and *ℓ*1 are slightly lower than those obtained by BDSD. Note that BDSD achieved the third best general figures just below the proposed log and *ℓ*1 algorithms. With p/P=16 the proposed log algorithm provides the best results except for Q, Q4 and SAM values, where competitive values are obtained. The proposed log achieves a slightly lower Q value than PRACS and a slightly higher SAM value than Brovey. In general, PRACS is the second best performing method for this image for p/P=16. For the FORMOSAT-2 image in Figure 3c, the proposed *ℓ*1 and log algorithms obtained the best numerical results for a p/P=4 magnification. Both methods provide similar results, which are better than all the one provided by the competing methods. For a ratio p/P=16, there is not a clear winner. The proposed methods are competitive in this image although they do not stand out in any of the measures. Table 11 and Table 12 show, respectively, the QNR quantitative results for the SPOT-5 and the FORMOSAT-2 images in Figure 3 for the decimation ratios p/P=4 and p/P=16. In Table 11, EXP achieves the best $D_{S}$, $D_{\lambda }$ and QNR scores. In this table, the proposed methods obtain good scores. The log method obtains the second best $D_{\lambda }$ and $D_{S}$ values and very high QNR values for both decimation ratios. Results for the FORMOSAT image, shown in Table 12, are very similar although in this case, BDSD obtains the best $D_{S}$ and QNR values for p/P=4 and ATWT-M3 for p/P=16.

Table 13 shows the required CPU time in seconds on a 2.40GHz Intel^®^ Xeon^®^ CPU for the pansharpening of a MS image with 4 bands to a 1024×1024 size, for p/P=4 and p/P=16, using the different methods under comparison. Equation (18) has been solved using the Conjugate Gradient method which required, to achieve convergence, less than 30 iterations for the *ℓ*1 prior and at least 1000 iterations for the log prior. This explains the differences between their required CPU time. Note that the proposed methods automatically estimate the model parameters which increases the running time but makes our methods parameter free.

### 6.2. Qualitative Comparison

**Figure 7:**
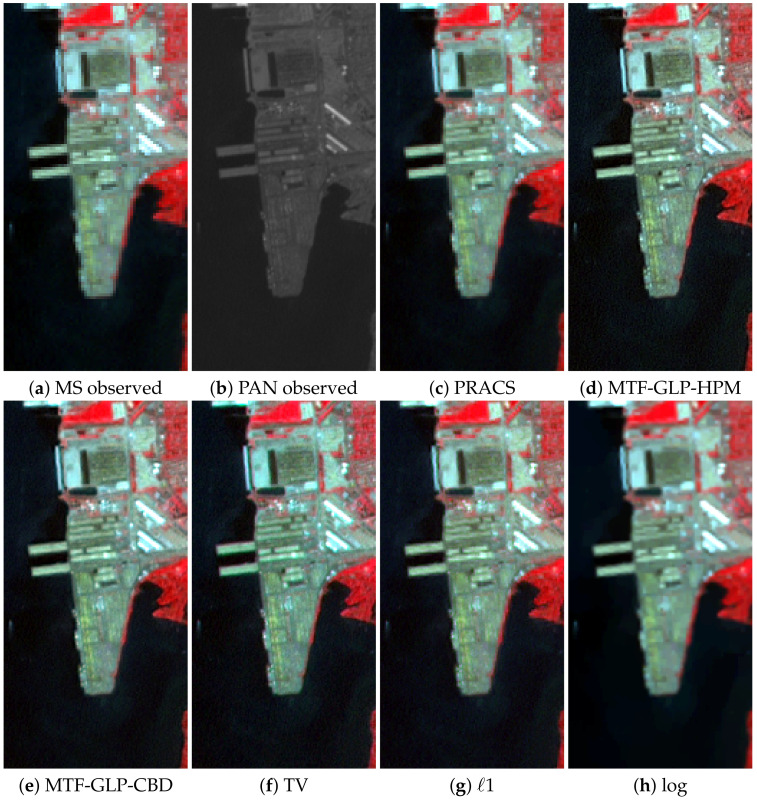
A region of interest of the LANDSAT 7-ETM+ Chesapeake Bay image in Figure 1a. Observed images: (**a**) 128×64 MS, (**b**) 256×128 PAN. 256×128 pansharpened images by: (**c**) PRACS, (**d**) MTF-GLP-High Pass Modulation (HPM), (**e**) MTF-GLP-CBD, (**f**) TV, (**g**) *ℓ*1 and (**h**) log methods.

Figure 7 shows a small region of interest of the observed LANDSAT-7 images in Figure 1 and the pansharpening results with p/P=4 obtained by the proposed methods and the competing ones with the best quantitative performance, that is, PRACS, MTF-GLP-HPM, MTF-GLP-CBD and TV methods. All color images in this figure are RGB images formed from the B4, B3 and B2 Landsat bands. Since we are using full resolution images, there is no ground truth to compare with, so a visual analysis of the resulting images is performed. The improved resolution of all the pansharpening results in Figure 7c–h with respect to the observed MS image in Figure 7a is evident. PRACS, MTF-GLP-HPM and MTF-GLP-CBD images in Figure 7c–e have a lower detail level than TV and the proposed *ℓ*1 method, see Figure 7f,g, respectively. See, for instance, the staircase effects in some diagonal edges not present in the TV and proposed *ℓ*1 method results. The PRACS, MTF-GLP-HPM and MTF-GLP-CBD methods produce similar, but lower, spectral quality than the proposed method, which is consistent with the numerical results in Table 3 and discussion presented in Section 6.1. The image obtained using the *ℓ*1 method, Figure 7g, has colors closer to those of the observed MS image than the TV image, Figure 7f, which is also somewhat noisier. The log method is very good at removing noise in the image (see the sea area) but it tends to remove other fine details too.

**Figure 8:**
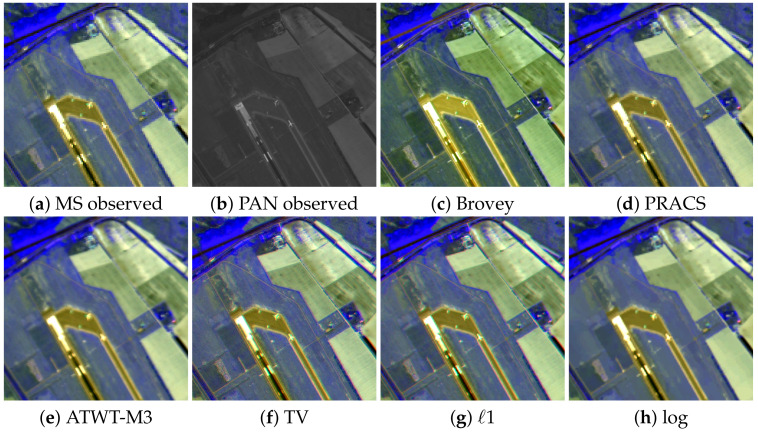
A region of interest of the SPOT-5 Roma image in Figure 3a. Observed images: (**a**) 128×128 MS, (**b**) 512×512 PAN. 512×512 pansharpened images by: (**c**) Brovey, (**d**) PRACS, (**e**) Additive A Trous Wavelet Transform (ATWT)-M3, (**f**) TV, (**g**) *ℓ*1 and (**h**) log methods.

Figure 8 shows a region of interest of the observed SPOT-5 images in Figure 3a,b and the pansharpening results with p/P=16 obtained using the competing methods with the best performance on this image, that is, Brovey, PRACS, ATWT-M3, and TV methods, and the proposed *ℓ*1 and log methods. All color images in this figure are RGB images formed from the bands B3, B2 and B1 of the SPOT-5 image. The Brovey method (Figure 8c) produces the highest spectral distortion, however, it also recovers more spatial details in the image (see the airport runway and plane). ATWT-M3 (Figure 8e), on the other hand, produces a blurry image. PRACS produces a sharper image, see Figure 8d, but details in the PAN image do not seem to be well-integrated. The TV method (Figure 8f) and the proposed *ℓ*1 and log methods (Figure 8g,h obtain the most consistent results, with high spatial details and low spectral distortion. However, TV introduces staircase artifacts on diagonal lines that are not noticeable in the *ℓ*1 and log images. As with the LANDSAT-7 image, the log image in Figure 8h lacks some small details, removed by the method along with noise.

## 7. Conclusions

A variational Bayesian methodology for the pansharpening problem has been proposed. In this methodology, we model the relation between the MS high resolution image and the PAN image as a linear combination of the MS bands whose weights are estimated from the available data. The observed MS image is modelled as a downsampled version of the original MS image. The expected characteristics of the pansharpened image are incorporated in the form of SG sparse image priors. Two penalty functions corresponding to SG distributions are used, *ℓ*1 and log. All the unknowns and model parameters have been automatically estimated within the variational Bayesian modelling and inference, and an efficient algorithm has been obtained.

The proposed *ℓ*1 and log methods have been compared to classic and state-of-the-art methods obtaining very good results both quantitative and qualitatively. In general, they have obtained the best quantitative results for LANDSAT-7 ETM+, SPOT-5 and FORMOSAT-2 images with a resolution ratio of 4 and SPOT-5 with a resolution ratio of 16. Competitive results were also obtained for the FORMOSAT-2 image with a resolution ratio of 16. They stand out in terms of spectral consistency while improving the spatial resolution of pansharpened images. We argue that the superior spectral consistency of SG methods arises from the modelling of the PAN image which selectively incorporates PAN detailed information into the different MS high resolution bands without changing their spectral properties. Qualitatively, SG methods produce results consistent with the observed PAN and MS images and with the numerical results previously described. The log method is better at removing noise in the images, at the cost of removing some fine details.

## Figures and Tables

**Table 1:** Some possible penalty functions.

Label	ρ(s)	$\rho^{\prime }\left(s\right)/\left|s\right|$
$\ell_{p}$, 0<p≤1	$\frac{1}{p}{\left|s\right|}^{p}$	${\left|s\right|}^{p-2}$
log	log(ϵ+|s|)	${\left(\epsilon +|s|\right)}^{-1}{\left|s\right|}^{-1}$

**Table 2:** Estimated λ values for the different sensors.

Sensor	Image	B1	B2	B3	B4	B5	B6
LANDSAT 7-ETM+	Figure 1	0.0986	0.1011	0.2576	0.5427	0	0
LANDSAT 7-ETM+	Figure 2a	0.0183	0.4243	0.0576	0.4998	0	0
LANDSAT 7-ETM+	Figure 2c	0	0.2283	0.1611	0.6106	0	0
SPOT-5	Figure 3a	0	0.2993	0.6897	0.0110	-	-
FORMOSAT-2	Figure 3c	0.0384	0.5566	0	0.4051	-	-

**Table 3:** Quantitative results on the LANDSAT 7-ETM+ Chesapeake Bay image. Bold values indicate the best score.

Method	Q8	Q	SAM	ERGAS	SCC
**EXP**	0.8335	0.8383	2.0223	5.1113	0.8718
**PCA**	0.7830	0.8074	2.7937	5.8086	0.8569
**IHS**	0.6795	0.6734	2.6640	7.8586	0.8223
**Brovey**	0.6798	0.6790	2.1605	7.5226	0.8148
**BDSD**	0.7599	0.7829	2.4662	5.9244	0.8539
**GS**	0.7447	0.7401	3.3052	8.7258	0.8040
**GSA**	0.8010	0.8202	2.4033	5.0688	0.8764
**PRACS**	0.8363	0.8423	2.0998	4.8655	0.8774
**HPF**	0.8137	0.8243	2.2221	5.1279	0.8834
**SFIM**	0.8008	0.8184	2.1857	5.0228	0.8905
**Indusion**	0.8052	0.8167	2.2906	5.5412	0.8495
**ATWT**	0.8100	0.8208	2.3482	5.3642	0.8809
**AWLP**	0.8267	0.8315	2.2516	5.3873	0.8734
**ATWT-M2**	0.7737	0.7802	2.6004	5.8133	0.8317
**ATWT-M3**	0.7884	0.7925	2.5720	5.8973	0.8367
**MTF-GLP**	0.8157	0.8258	2.2418	5.1307	0.8838
**MTF-GLP-HPM**	0.8037	0.8206	2.1878	5.0236	0.8918
**MTF-GLP-CBD**	0.8200	0.8292	2.2499	5.1015	0.8809
**TV**	0.8492	0.8606	2.1362	4.2505	0.9163
ℓ1	**0.8595**	**0.8694**	**1.8518**	**4.0954**	**0.9220**
**log**	0.7951	0.7856	1.8839	4.4819	0.9007

**Table 4:** QNR Quantitative results on the LANDSAT 7-ETM+ Chesapeake Bay image. Bold values indicate the best score.

	$D_{\lambda }$	$D_{S}$	QNR
**EXP**	0.0096	**0.0166**	**0.9740**
**PCA**	0.0555	0.1459	0.8066
**IHS**	0.1049	0.2888	0.6366
**Brovey**	0.0963	0.2290	0.6968
**BDSD**	0.1055	0.1486	0.7616
**GS**	0.0716	0.1992	0.7435
**GSA**	0.0605	0.1167	0.8298
**PRACS**	0.0154	0.0823	0.9035
**HPF**	0.0857	0.1308	0.7947
**SFIM**	0.0834	0.1146	0.8116
**Indusion**	0.0595	0.0525	0.8911
**ATWT**	0.1044	0.1500	0.7612
**AWLP**	0.1045	0.1517	0.7596
**ATWT-M2**	0.1530	0.2327	0.6499
**ATWT-M3**	0.1227	0.1975	0.7041
**MTF-GLP**	0.0917	0.1378	0.7831
**MTF-GLP-HPM**	0.0890	0.1215	0.8004
**MTF-GLP-CBD**	0.0585	0.1081	0.8397
**TV**	0.0425	0.0919	0.8695
**l1**	0.0338	0.0527	0.9153
**log**	**0.0090**	0.0364	0.9549

**Table 5:** Quantitative results using Wald’s protocol on the LANDSAT 7-ETM+ Netherland image in Figure 2a. Bold values indicate the best score.

	Q8	Q	SAM	ERGAS	SCC
**EXP**	0.4727	0.8849	3.0445	7.7235	0.8686
**PCA**	0.3759	0.7617	3.8165	12.6420	0.8205
**IHS**	0.3322	0.7479	1.7633	10.7598	0.8570
**Brovey**	0.2892	0.7675	**0.0000**	10.7809	0.8492
**BDSD**	0.7205	0.9520	1.7296	4.6748	0.9777
**GS**	0.3860	0.7833	3.3486	11.7850	0.8323
**GSA**	0.5543	0.8474	2.5086	10.2990	0.8707
**PRACS**	**0.8230**	**0.9720**	0.9558	**2.9097**	**0.9878**
**HPF**	0.6420	0.9045	2.0699	7.4387	0.9458
**SFIM**	0.5950	0.9043	1.8898	8.1778	0.9379
**Indusion**	0.4108	0.8406	3.6963	9.6521	0.8269
**ATWT**	0.5582	0.8741	2.6859	9.6507	0.9267
**AWLP**	0.4715	0.8741	2.2059	10.1057	0.9195
**ATWT-M2**	0.3943	0.8436	3.7879	8.7289	0.8606
**ATWT-M3**	0.4861	0.8685	3.5274	7.7506	0.8829
**MTF-GLP**	0.5975	0.8946	2.2544	8.1279	0.9351
**MTF-GLP-HPM**	0.5659	0.8926	2.0133	9.0221	0.9272
**MTF-GLP-CBD**	0.6095	0.9001	2.1726	7.8290	0.9392
**TV**	0.4798	0.8906	3.4873	7.1207	0.8977
**l1**	0.4815	0.9044	3.3783	6.9422	0.9022
**log**	0.4931	0.8920	2.9187	6.9321	0.8952

**Table 6:** Quantitative results using Wald’s protocol on the LANDSAT 7-ETM+ Netherland image in Figure 2c. Bold values indicate the best score.

	Q8	Q	SAM	ERGAS	SCC
**EXP**	0.7874	0.7867	3.3362	5.9279	0.8521
**PCA**	0.6167	0.4854	5.7078	12.8127	0.5788
**IHS**	0.5343	0.3778	4.7417	12.9526	0.5918
**Brovey**	0.5465	0.4063	3.4605	13.0876	0.5960
**BDSD**	0.7575	0.7733	3.9348	6.8610	0.7856
**GS**	0.5899	0.4369	6.1158	13.9275	0.5522
**GSA**	0.7323	0.7444	4.0920	7.2078	0.7535
**PRACS**	0.7822	0.7829	3.4787	6.3244	0.8037
**HPF**	0.7167	0.7241	3.8135	7.3266	0.7603
**SFIM**	0.6908	0.7181	4.6693	9.0262	0.7261
**Indusion**	0.7230	0.7346	3.8229	7.2177	0.7631
**ATWT**	0.6948	0.6971	4.0402	8.0306	0.7240
**AWLP**	0.7110	0.7066	3.8124	8.1733	0.7165
**ATWT-M2**	0.6332	0.6288	4.5223	8.5542	0.6135
**ATWT-M3**	0.6993	0.6967	4.3028	7.4420	0.7201
**MTF-GLP**	0.7116	0.7172	3.8632	7.5134	0.7457
**MTF-GLP-HPM**	0.6875	0.7133	4.6780	9.1110	0.7161
**MTF-GLP-CBD**	0.7519	0.7595	3.7522	6.8698	0.7740
**TV**	0.7843	0.8065	3.8402	5.7351	0.8519
**l1**	**0.8118**	**0.8196**	3.1337	**5.1831**	**0.8853**
**log**	0.7750	0.7682	**3.0810**	5.3115	0.8818

**Table 7:** QNR quantitative results on the LANDSAT 7-ETM+ Netherland image in Figure 2a. Bold values indicate the best score.

	$D_{\lambda }$	$D_{S}$	QNR
**EXP**	**0.0104**	0.0593	0.9309
**PCA**	0.2463	0.3998	0.4523
**IHS**	0.2632	0.4035	0.4394
**Brovey**	0.2182	0.3873	0.4790
**BDSD**	0.0159	0.0505	**0.9344**
**GS**	0.2335	0.4067	0.4548
**GSA**	0.2139	0.3240	0.5314
**PRACS**	0.0665	0.2106	0.7369
**HPF**	0.1711	0.2638	0.6102
**SFIM**	0.1610	0.2513	0.6282
**Indusion**	0.1354	0.1612	0.7252
**ATWT**	0.1968	0.2961	0.5654
**AWLP**	0.1977	0.2954	0.5653
**ATWT-M2**	0.1727	0.3127	0.5686
**ATWT-M3**	0.1187	0.2143	0.6924
**MTF-GLP**	0.1796	0.2791	0.5915
**MTF-GLP-HPM**	0.1688	0.2661	0.6100
**MTF-GLP-CBD**	0.1741	0.2778	0.5965
**TV**	0.0912	0.1127	0.8064
**l1**	0.0342	**0.0386**	0.9286
**log**	0.0157	0.0627	0.9225

**Table 8:** QNR quantitative results on the LANDSAT 7-ETM+ Netherland image in Figure 2c. Bold values indicate the best score.

	$D_{\lambda }$	$D_{S}$	QNR
**EXP**	**0.0067**	**0.0157**	**0.9778**
**PCA**	0.1893	0.5019	0.4038
**IHS**	0.2040	0.5973	0.3205
**Brovey**	0.1979	0.5227	0.3829
**BDSD**	0.0134	0.0107	0.9761
**GS**	0.1999	0.5237	0.3811
**GSA**	0.0986	0.2337	0.6907
**PRACS**	0.0255	0.1176	0.8599
**HPF**	0.1594	0.2772	0.6075
**SFIM**	0.1124	0.2272	0.6859
**Indusion**	0.1352	0.2054	0.6871
**ATWT**	0.1822	0.3234	0.5534
**AWLP**	0.1765	0.3101	0.5682
**ATWT-M2**	0.1903	0.3467	0.5290
**ATWT-M3**	0.0917	0.1581	0.7647
**MTF-GLP**	0.1656	0.2933	0.5897
**MTF-GLP-HPM**	0.1164	0.2424	0.6694
**MTF-GLP-CBD**	0.0854	0.1953	0.7359
**TV**	0.0381	0.1186	0.8478
**l1**	0.0228	0.0704	0.9084
**log**	0.0073	0.0199	0.9730

**Table 9:** Quantitative results using Wald’s protocol on the SPOT-5 Roma image. Bold values indicate the best score.

p/P	4					16				
**Method**	**Q4**	**Q**	**SAM**	**ERGAS**	**SCC**	**Q4**	**Q**	**SAM**	**ERGAS**	**SCC**
**EXP**	**0.8766**	**0.8859**	1.7048	3.7857	0.8640	**0.7325**	0.7407	2.5071	2.8441	0.6049
**PCA**	0.4067	0.5360	5.1646	12.3346	0.2788	0.3927	0.5091	5.7208	6.2911	0.2443
**IHS**	0.4072	0.5238	3.9951	12.2342	0.2772	0.3973	0.5051	4.4508	6.1770	0.2520
**Brovey**	0.4124	0.5337	1.8413	12.1960	0.2594	0.4019	0.5124	**2.4000**	6.1718	0.2482
**BDSD**	0.8559	0.8825	2.0565	4.2776	0.8235	0.5947	0.6231	3.0328	4.3050	0.2804
**GS**	0.4102	0.5364	5.0523	12.1272	0.2634	0.3985	0.5124	5.5849	6.1471	0.2405
**GSA**	0.4897	0.5384	2.9893	11.0363	0.1932	0.4997	0.5354	3.1189	5.3703	0.2164
**PRACS**	0.8220	0.8380	2.0536	4.8936	0.7298	0.7291	**0.7458**	2.5190	2.9647	0.5295
**HPF**	0.7488	0.7695	2.0632	6.3388	0.6250	0.5888	0.6124	2.8370	4.5969	0.2988
**SFIM**	0.7744	0.7860	1.9658	6.0694	0.6447	0.6052	0.6232	2.6792	4.4425	0.3079
**Indusion**	0.7473	0.7894	2.1609	5.9717	0.6761	0.5301	0.5935	3.5689	4.9656	0.3408
**ATWT**	0.6928	0.7171	2.2358	7.6433	0.5183	0.5849	0.6092	2.8932	4.7245	0.3099
**AWLP**	0.7066	0.7260	2.1798	7.6246	0.5075	0.5947	0.6173	2.8504	4.6963	0.3052
**ATWT-M2**	0.7229	0.7343	2.3084	6.3145	0.4678	0.6723	0.6822	2.7291	3.3277	0.3965
**ATWT-M3**	0.7837	0.7930	2.2394	5.1935	0.6714	0.6924	0.7019	2.7026	3.1094	0.4801
**MTF-GLP**	0.7289	0.7507	2.1201	6.7975	0.5766	0.5775	0.6023	2.9275	4.8355	0.3022
**MTF-GLP-HPM**	0.7553	0.7675	2.0005	6.4923	0.5972	0.5928	0.6111	2.7388	4.7066	0.3073
**MTF-GLP-CBD**	0.7718	0.7870	2.0188	6.0490	0.6215	0.6021	0.6217	2.7767	4.5632	0.3118
**TV**	0.7472	0.7893	3.1882	6.1393	0.6162	0.6480	0.6872	3.5109	3.9763	0.3265
ℓ1	0.8617	0.8783	2.0688	4.1557	0.8196	0.6409	0.7017	3.7793	3.7117	0.3792
**log**	0.8636	0.8762	**1.6053**	**3.3673**	**0.8923**	0.7323	0.7395	2.4228	**2.7072**	**0.6262**

**Table 10:** Quantitative results using Wald’s protocol on the FORMOSAT-2 Salon-de-Provence image. Bold values indicate the best score.

p/P	4					16				
**Method**	**Q4**	**Q**	**SAM**	**ERGAS**	**SCC**	**Q4**	**Q**	**SAM**	**ERGAS**	**SCC**
**EXP**	0.8610	0.8617	1.8158	3.7257	0.8460	0.6918	0.6925	2.5057	2.6934	0.5906
**PCA**	0.7858	0.8089	2.2098	5.0711	0.6595	0.7415	0.7668	2.6373	2.7485	0.5870
**IHS**	0.7912	0.8159	1.9637	4.6293	0.6695	0.7445	0.7720	2.4527	2.5803	0.5894
**Brovey**	0.7861	0.8146	1.9106	4.5332	0.6644	0.7385	0.7687	2.4345	2.5597	0.5793
**BDSD**	0.8443	0.8545	2.0292	4.2608	0.7232	0.7701	0.7823	2.5460	2.7217	0.5821
**GS**	0.7979	0.8195	2.1375	4.9193	0.6619	0.7506	0.7753	2.5761	2.6860	0.5888
**GSA**	0.7859	0.8167	2.1855	5.2508	0.6508	0.7607	0.7772	2.5510	2.7698	0.5796
**PRACS**	0.8495	0.8592	1.9581	4.0842	0.7373	**0.7947**	**0.8016**	2.4240	**2.3683**	0.6230
**HPF**	0.8469	0.8523	1.9799	4.2210	0.7782	0.7813	0.7885	2.4609	2.5388	0.5900
**SFIM**	0.8470	0.8523	1.9821	4.2718	0.7755	0.7817	0.7888	2.4600	2.5658	0.5874
**Indusion**	0.8304	0.8371	2.0075	4.4361	0.7407	0.7519	0.7698	2.4986	2.6941	0.5758
**ATWT**	0.8391	0.8449	2.0374	4.5675	0.7663	0.7901	0.7965	2.4679	2.5399	0.6161
**AWLP**	0.8408	0.8473	1.8896	4.3549	0.7671	0.7909	0.7979	**2.3689**	2.4540	0.6150
**ATWT-M2**	0.8277	0.8326	2.2307	4.2164	0.6975	0.7672	0.7704	2.5499	2.3709	0.6325
**ATWT-M3**	0.8325	0.8356	2.2137	4.0586	0.7341	0.7639	0.7660	2.5659	2.3878	**0.6384**
**MTF-GLP**	0.8477	0.8533	1.9883	4.2705	0.7704	0.7901	0.7968	2.4714	2.5563	0.6176
**MTF-GLP-HPM**	0.8476	0.8532	1.9889	4.3313	0.7676	0.7904	0.7971	2.4666	2.5937	0.6158
**MTF-GLP-CBD**	0.8493	0.8548	2.0076	4.2575	0.7730	0.7846	0.7916	2.5270	2.6394	0.6123
**TV**	0.8696	0.8790	2.1437	3.7602	0.7876	0.7807	0.7878	2.7906	2.4374	0.5956
ℓ1	**0.8946**	**0.8974**	1.9200	**3.3526**	0.8457	0.7691	0.7625	3.0726	2.6041	0.5503
**log**	0.8706	0.8683	**1.7597**	3.3686	**0.8751**	0.6889	0.6848	2.4975	2.6009	0.6050

**Table 11:** QNR Quantitative results on the SPOT-5 Roma image. Bold values indicate the best score.

p/P	4			16		
	$D_{\lambda }$	$D_{S}$	**QNR**	$D_{\lambda }$	$D_{S}$	**QNR**
**EXP**	**0.0041**	**0.0150**	**0.9809**	**0.0001**	**0.0312**	**0.9687**
**PCA**	0.2047	0.4094	0.4697	0.3094	0.5035	0.3429
**IHS**	0.2389	0.4158	0.4447	0.3574	0.5143	0.3121
**Brovey**	0.1804	0.3799	0.5082	0.2890	0.4754	0.3730
**BDSD**	0.0108	0.0922	0.8980	0.0388	0.0344	0.9281
**GS**	0.1964	0.4100	0.4741	0.3045	0.5044	0.3447
**GSA**	0.2194	0.3421	0.5135	0.3267	0.4287	0.3846
**PRACS**	0.0325	0.1555	0.8171	0.0656	0.2162	0.7324
**HPF**	0.0851	0.1405	0.7864	0.2149	0.2556	0.5844
**SFIM**	0.0661	0.1256	0.8167	0.1949	0.2416	0.6107
**Indusion**	0.0580	0.0370	0.9072	0.2458	0.1587	0.6345
**ATWT**	0.1310	0.2119	0.6849	0.2398	0.3037	0.5293
**AWLP**	0.1030	0.1950	0.7221	0.2015	0.2814	0.5738
**ATWT-M2**	0.0728	0.2002	0.7416	0.0996	0.1691	0.7482
**ATWT-M3**	0.0162	0.0349	0.9494	0.0493	0.0328	0.9195
**MTF-GLP**	0.1040	0.1586	0.7539	0.2511	0.3000	0.5242
**MTF-GLP-HPM**	0.0858	0.1441	0.7825	0.2314	0.2868	0.5481
**MTF-GLP-CBD**	0.0657	0.1272	0.8154	0.1922	0.2681	0.5912
**TV**	0.3399	0.1830	0.5394	0.1866	0.1510	0.6906
**l1**	0.0277	0.0378	0.9356	0.1927	0.1500	0.6862
**log**	0.0056	0.0272	0.9674	0.0422	0.0380	0.9214

**Table 12:** QNR quantitative results on the FORMOSAT-2 Salon-de-Provence image. Bold values indicate the best score.

p/P	4			16		
	$D_{\lambda }$	$D_{S}$	**QNR**	$D_{\lambda }$	$D_{S}$	**QNR**
**EXP**	**0.0087**	0.0837	0.9083	**0.0086**	0.0931	0.8990
**PCA**	0.1108	0.2190	0.6945	0.1503	0.3083	0.5877
**IHS**	0.1083	0.2127	0.7020	0.1508	0.3027	0.5921
**Brovey**	0.0859	0.1964	0.7345	0.1215	0.2815	0.6312
**BDSD**	0.0179	**0.0150**	**0.9673**	0.0264	0.1775	0.8008
**GS**	0.0970	0.2084	0.7149	0.1366	0.2971	0.6069
**GSA**	0.1254	0.2095	0.6914	0.1618	0.2963	0.5899
**PRACS**	0.0621	0.1656	0.7826	0.0878	0.2413	0.6921
**HPF**	0.0896	0.1130	0.8075	0.1147	0.1559	0.7473
**SFIM**	0.0877	0.1121	0.8101	0.1128	0.1556	0.7492
**Indusion**	0.0510	0.0174	0.9325	0.0938	0.0965	0.8187
**ATWT**	0.1227	0.1529	0.7432	0.1355	0.1951	0.6958
**AWLP**	0.1290	0.1500	0.7404	0.1393	0.1887	0.6983
**ATWT-M2**	0.1184	0.1550	0.7449	0.0967	0.1238	0.7915
**ATWT-M3**	0.0871	0.0951	0.8261	0.0586	**0.0388**	**0.9049**
**MTF-GLP**	0.1004	0.1277	0.7847	0.1437	0.1996	0.6854
**MTF-GLP-HPM**	0.0980	0.1269	0.7875	0.1410	0.1989	0.6881
**MTF-GLP-CBD**	0.0910	0.1222	0.7979	0.1317	0.1919	0.7017
**TV**	0.1114	0.0787	0.8186	0.0951	0.1811	0.7410
**l1**	0.0425	0.0514	0.9083	0.0594	0.0847	0.8609
**log**	0.0094	0.0917	0.8998	0.0174	0.1011	0.8832

**Table 13:** Elapsed CPU time in seconds for the different pansharpening methods on a 1024 × 1024 image and with different p/P ratios.

*P*	*p*	PCA	IHS	Brovey	BDSD	GS
512×512	1024×1024	0.9	0.04	0.04	0.9	0.4
256×256	1024×1024	0.3	0.03	0.03	0.8	0.3
***P***	***p***	GSA	PRACS	HPF	SFIM	Indusion
512×512	1024×1024	1	2.2	0.2	0.5	0.5
256×256	1024×1024	1.6	1.2	0.2	0.18	0.3
***P***	***p***	**ATWT**	**AWLP**	**ATWT-M2**	**ATWT-M3**	**MTF-GLP**
512×512	1024×1024	11	14	10	10	0.9
256×256	1024×1024	3	3	7	7	0.6
***P***	***p***	**MTF-HPM**	**MTF-CBD**	**TV**	*ℓ* **1**	**log**
512×512	1024×1024	0.8	2	1.5 $10^{3}$	808	1.1 $10^{4}$
256×256	1024×1024	0.6	0.6	1.6 $10^{3}$	3 $10^{3}$	2.8 $10^{4}$

## Abbreviations

The following abbreviations are used in this manuscript:

MS	multispectral
PAN	pancrhomatic
PCA	principal components analysis
IHS	intensity-hue-saturation
CS	component substitution
MRA	multi-resolution analysis
VO	variational optimization
DL	deep learning
CS	component substitution
GS	Gram-Schmidt
HPF	high-pass filtering
WT	wavelet transform
GLP	generalized Laplacian pyramid
NSCT	non-subsampled contourlet transform
AWT	“a trous” wavelet transforms
SFIM	smoothing filter based intensity modulation
DNN	deep neural networks
MSDA	modified sparse denoising autoencoder
CSDA	coupled sparse denoising autoencoder
PCDRN	progressive cascade deep residual network
GAN	generative adversarial network
SG	super-Gaussian
Brovey	Brovey transform
BDSD	band-dependent spatial-detail
GSA	Gram-Schmidt adaptive
PRACS	partial replacement adaptive component substitution
ATWT	additive “a trous” wavelet transform
ATLP	additive wavelet luminance proportional
MTF	modulation transfer functions
HPM	high pass modulation
CBD	context based decision
TV	total variation
UQI	universal quality index
SCC	spatial correlation coefficient
SAM	spectral angle mapper
ERGAS	erreur relative globale adimensionnelle de synthese
QNR	quality with no reference
